# Kaposi Sarcoma Presenting as Hemophagocytic Lymphohistiocytosis Mimicking Infection and Lymphoma: A Diagnostic Challenge

**DOI:** 10.7759/cureus.105780

**Published:** 2026-03-24

**Authors:** Vahit C Cavdar, Çağdaş Kaya, Ayli Heydari, Chalil C Chatzi Chasan, Ayse Satilmisoglu, Yalçın Gökmen, Feray Akbas, Gülhan Özdemir, Gülben Erdem Huq, Esra S Kaya, Zeynep T Dincer, Ilkay Gulturk, Kadir Karismaz, Sevil Sadri

**Affiliations:** 1 Department of Internal Medicine, University of Health Sciences, Istanbul Training and Research Hospital, Istanbul, TUR; 2 Department of Infectious Disease, University of Health Sciences, Istanbul Training and Research Hospital, İstanbul, TUR; 3 Department of Pathology, University of Health Sciences, Istanbul Training and Research Hospital, Istanbul, TUR; 4 Department of Dermatology, University of Health Sciences, Istanbul Training and Research Hospital, Istanbul, TUR; 5 Department of Rheumatology, University of Health Sciences, Istanbul Training and Research Hospital, Istanbul, TUR; 6 Department of Medical Oncology, University of Health Sciences, Istanbul Training and Research Hospital, Istanbul, TUR; 7 Department of Hematology, University of Health Sciences, Istanbul Training and Research Hospital, Istanbul, TUR

**Keywords:** fever, hemophagocytic lymphohistiocytosis, hepatitis b, hepatomegaly, kaposi sarcoma, splenomegaly

## Abstract

Kaposi sarcoma (KS) is an angioproliferative malignancy associated with human herpesvirus 8 and typically presents with cutaneous lesions. Visceral involvement and presentation as secondary hemophagocytic lymphohistiocytosis (HLH) are rare and may obscure the underlying diagnosis.

A 45-year-old woman with hepatitis B carrier status and compensated cirrhosis presented with dizziness, fever, rash, fatigue, cytopenias, and hepatosplenomegaly. Laboratory evaluation revealed normocytic anemia, thrombocytopenia, elevated transaminases, and markedly increased inflammatory markers, prompting hospitalization for suspected infection and hematologic disease. Despite broad antimicrobial therapy and extensive investigations, her condition progressed with persistent inflammation, raising the suspicion of secondary HLH. Empirical treatment with etoposide and dexamethasone was initiated. Positron emission tomography demonstrated hypermetabolic bilateral axillary lymphadenopathy, and excisional biopsy of the right axillary lymph node revealed KS. The patient was transferred to oncology care, where therapy was changed to etoposide plus paclitaxel with clinical stabilization.

KS presenting as secondary HLH is extremely uncommon and poses significant diagnostic challenges, particularly in the absence of typical cutaneous findings. This case underscores the importance of considering occult malignancy in patients with fever, cytopenias, and hepatosplenomegaly who do not respond to conventional therapy. Timely tissue diagnosis is crucial in patients with suspected HLH when the underlying cause is unclear, as the early identification of malignancy can significantly alter management and improve outcomes.

## Introduction

Kaposi sarcoma (KS) is a disease associated with human herpesvirus 8 infection, also known as KS-associated herpesvirus, and may lead to life-threatening complications. KS typically presents on the skin as isolated or multiple violaceous macules, papulonodular lesions, or plaques; however, in some cases, it may manifest with atypical presentations [1,2].

In rare situations, malignancies may also trigger hemophagocytic lymphohistiocytosis (HLH), a severe hyperinflammatory syndrome characterized by uncontrolled immune activation and cytokine release. Epidemiological data from a population-based study in Sweden reported that the annual incidence of malignancy-associated HLH is ≥0.62 per 100,000 adults and affects ≥0.6% of all hematological malignancies, highlighting the clinical importance of recognizing malignancy as an underlying trigger of HLH [3].

When KS was first described, it was defined as pigmented, violaceous skin tumors seen predominantly in elderly men. This form is classified as classic Kaposi, or Mediterranean-type Kaposi, because it is frequently observed in countries surrounding the Mediterranean basin. The second form is the African type of Kaposi, seen in sub-Saharan African countries. In this form, males are also predominant, although it can occur in childhood as well. The third form of KS emerged after transplantation, particularly in patients who underwent solid organ transplantation; Kaposi cases reported in this group raised the possibility of an association between the disease and immunosuppression, and it has therefore been defined as the immunosuppression-related form. The fourth epidemiological form is AIDS-associated Kaposi. More recently, a fifth epidemiological form has been described in homosexual men who are not infected with HIV [4].

In this case, we aimed to emphasize that KS, although rare, should also be considered among the possible etiologies of fever of unknown origin (FUO).

## Case presentation

The patient was a 45-year-old woman with a known history of hepatitis B carrier status under regular follow-up and compensated cirrhosis not requiring medication. She presented to the emergency department with dizziness, fever, rash, nausea, and fatigue that had begun one week prior to admission, accompanied by the progression of the rash and its spread over the entire body. One week before the presentation, she had used clindamycin for tonsillitis.

On initial physical examination, a generalized blanching maculopapular rash sparing the face was present over the entire body, reportedly developing after intramuscular ceftriaxone was administered at an outside center. Hepatosplenomegaly was noted, while other system examinations were unremarkable. The patient was hospitalized for further evaluation of anemia and thrombocytopenia. Laboratory tests performed in the emergency department revealed normocytic anemia, an increase in aspartate aminotransferase (AST) and alanine aminotransferase (ALT) levels, and thrombocytopenia. Laboratory findings at the time of admission are presented in Table 1.

**Table 1:** Laboratory results at emergency department presentation and infectious disease-related investigations requested at the time of hospitalization. WBC: white blood cell (count); AST: aspartate aminotransferase; ALT: alanine aminotransferase; GGT: gamma-glutamyl transferase; ALP: alkaline phosphatase; PT: prothrombin time; INR: international normalized ratio; aPTT: activated partial thromboplastin time; CRP: C-reactive protein; HBsAg: hepatitis B surface antigen; anti-HBc IgM: hepatitis B core antibody immunoglobulin M; anti-HBc IgG: hepatitis B core antibody immunoglobulin G; HBV DNA: hepatitis B virus deoxyribonucleic acid; PCR: polymerase chain reaction; anti-HDV: hepatitis D virus antibody (delta antibody); anti-HEV IgM: hepatitis E virus antibody immunoglobulin M; anti-HCV: hepatitis C virus antibody; anti-HIV: human immunodeficiency virus antibody; EBV: Epstein-Barr virus; VCA: viral capsid antigen; HSV: herpes simplex virus; CMV: cytomegalovirus; IgM: immunoglobulin M; IgG: immunoglobulin G; IFAT: indirect fluorescent antibody test; *Leishmania* spp.: *Leishmania* species; MAT: microscopic agglutination test; T-SPOT.TB: T-SPOT tuberculosis test; IGRA: interferon-gamma release assay; VDRL: venereal disease research laboratory test; RPR: rapid plasma reagin test; COI: cut-off index

Investigation	Result	Reference range
WBC	8.32×10⁹/L	4-10×10⁹/L
Hemoglobin	7.5 g/dL	11-15 g/dL
Platelet count	84×10⁹/L	100-400×10⁹/L
Glucose	116 mg/dL	70-100 mg/dL
Urea	24.8 mg/dL	17-43 mg/dL
Creatinine	0.78 mg/dL	0.67-1.17 mg/dL
AST	75 U/L	0-50 U/L
ALT	46 U/L	0-50 U/L
GGT	19 U/L	0-55 U/L
ALP	53 U/L	43-115 U/L
Sodium	135 mmol/L	136-146 mmol/L
Potassium	3.71 mmol/L	3.5-5.1 mmol/L
PT (INR)	1.5	0.80-1.20
APTT	27.7 seconds	21-35 seconds
D-dimer	2.07 ng/mL	<500 ng/mL
Procalcitonin	4.98 µg/L	0-0.5 µg/L
Ferritin	752 ng/mL	24-336 ng/mL for men; 11-307 ng/mL for women
CRP	122 mg/L	0-5 mg/L
HBsAg	Positive (2612.85 COI)	<1 COI
Anti-HBc IgM	Negative	<1 COI
Anti-HBc IgG	Positive (3.91)	<1 COI
HBV DNA (quantitative PCR)	70.1 IU/mL (detectable)	<10 IU/mL
Delta antibody	Negative	<1 COI
Anti-HEV IgM	Negative	<1 COI
Anti-HCV	Negative	<1 COI
Anti-HIV	Negative	<1 COI
EBV VCA IgM	Negative	<35.9 U/mL
EBV VCA IgG	Positive	<17.9 U/mL
HSV type 1/2 IgM	Positive (25.9 U/mL)	<1 Index
CMV DNA PCR	Not detected	<200 IU/mL
CMV IgM	Negative	<30 U/mL
Measles IgM antibody	Negative	<0.9 index
Mumps IgM antibody	Negative	<0.9 index
Anti-rubella IgM	Negative	<0.75 COI
Toxoplasma IgM antibody	Negative	<1 COI
Parvo B19 IgM	Negative	<1 COI
Borrelia burgdorferi IgM	Negative	<1 COI
Brucella Rose Bengal	Negative	-
VDRL-RPR	Negative	-
Leishmania spp. IgG dipstick	Weakly positive	-
Leishmania spp. IgG (IFAT)	1/160 positive	<1/64
Leishmania spp. PCR	Negative	-
Leptospira (MAT)	Negative	<1:100
Weil-Felix test	Negative	<1/80
T-SPOT.TB (IGRA) test	Negative	≤4
Blood culture	Methicillin-resistant Staphylococcus caprae	-
Repeat blood culture	Diphtheroid growth	-
Sputum microscopy	>25 leukocytes/field; mixed flora	-

Following admission to the internal medicine ward, evaluation was planned to investigate the etiology of bicytopenia, the causes of hepatosplenomegaly, the elevated acute-phase reactants, and the cause of the rash. On the first day of hospitalization, the patient was consulted by the infectious diseases department due to elevated white blood cell count and procalcitonin levels, and moxifloxacin 400 mg once daily was initiated upon recommendation.

On the second day of hospitalization, a peripheral blood smear was requested by the hematology department, and upper and lower gastrointestinal endoscopy was planned to rule out gastrointestinal bleeding as a cause of anemia. No bleeding source or evidence of malignancy was detected on endoscopy and colonoscopy. No findings suggestive of hematological malignancy were detected on the patient's peripheral smear; thrombocytopenia was consistent with laboratory results, and normocytic anemia was observed.

**Figure 1:**
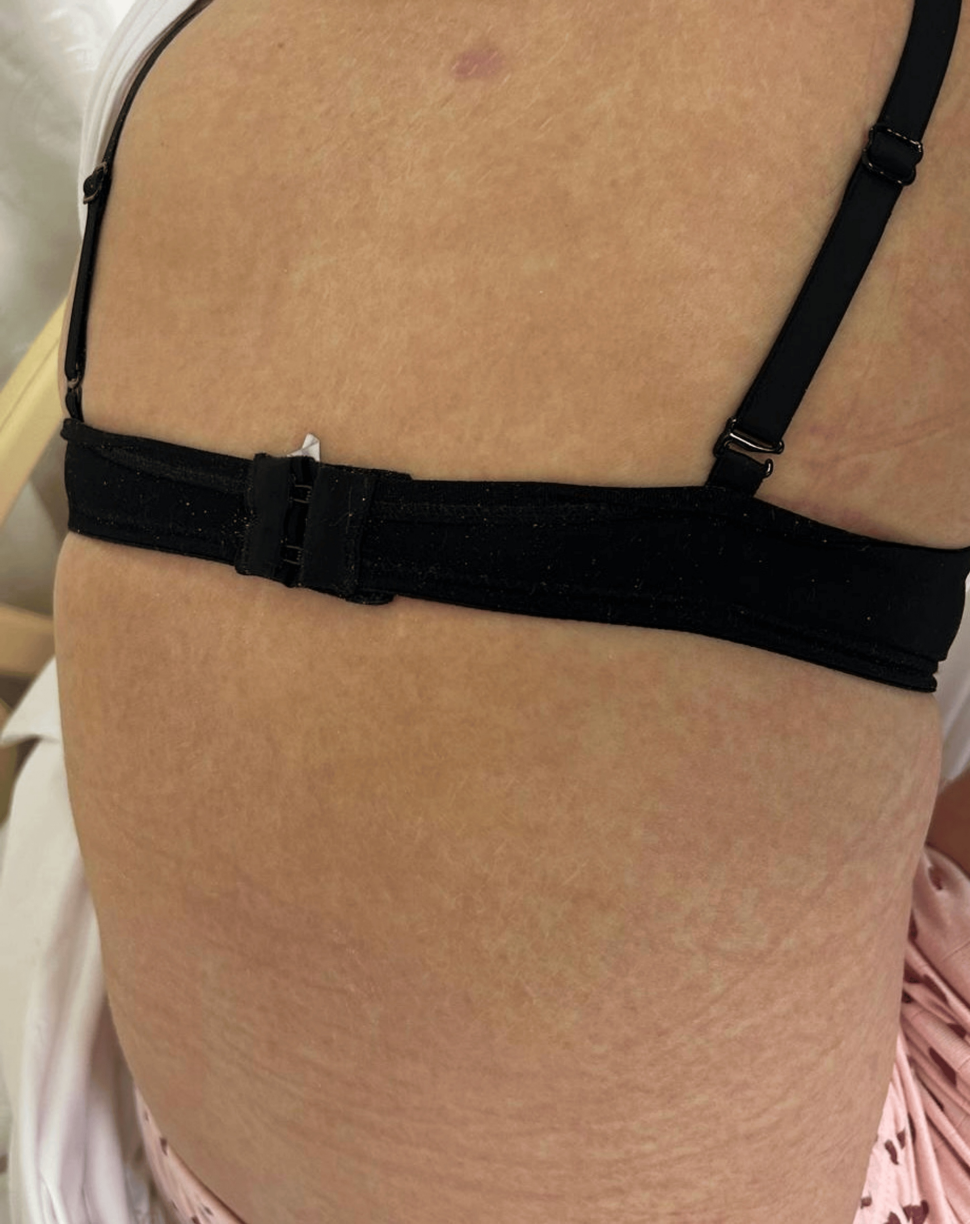
Rashes on the back.

**Figure 2:**
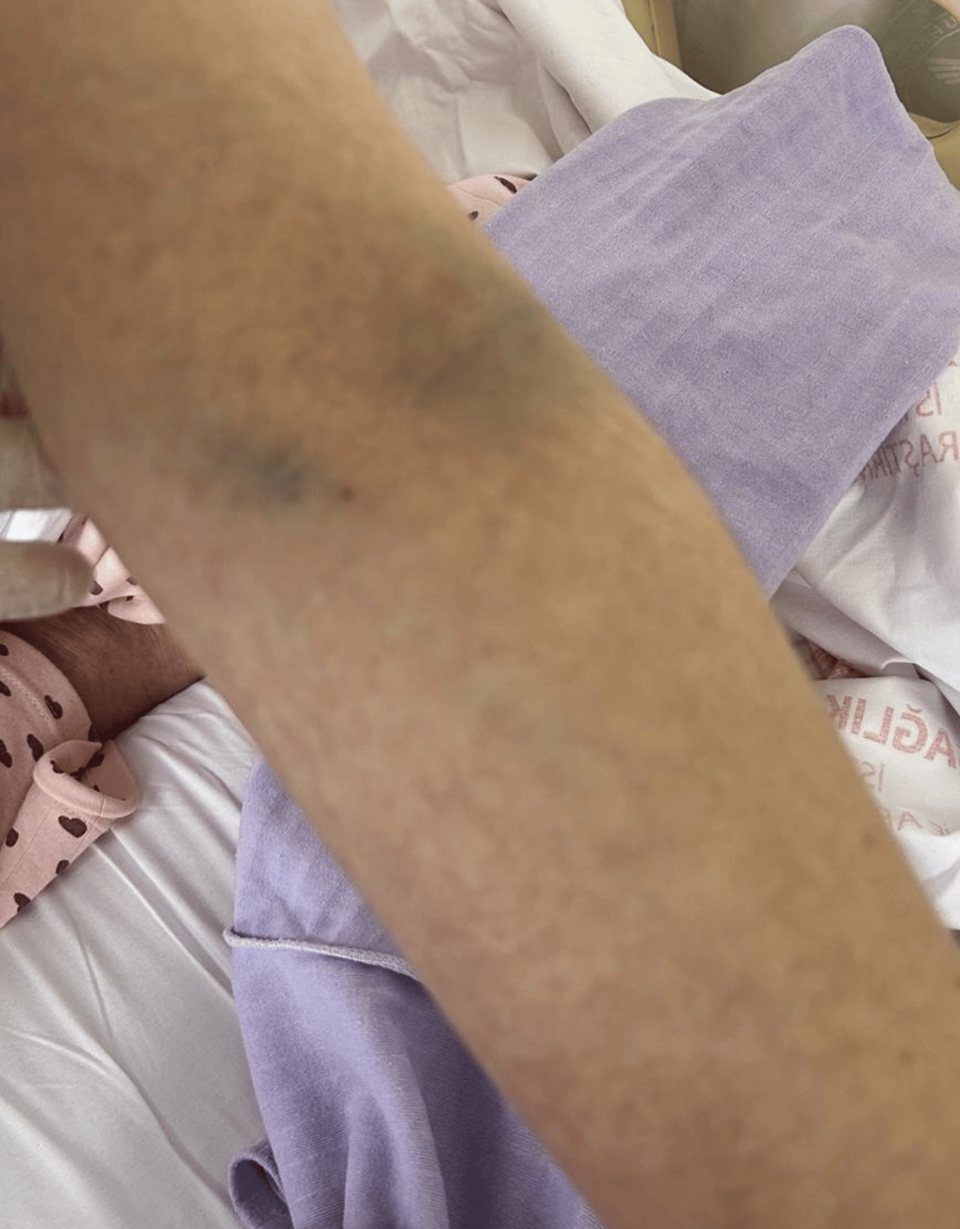
Rashes on the arm.

On the third day of hospitalization, QTc prolongation was detected on electrocardiography, leading to the discontinuation of moxifloxacin and initiation of doxycycline 100 mg orally twice daily. On the fourth day, gastroscopy was performed and revealed no pathological findings. During hospitalization, a dermatology consultation was obtained for the generalized maculopapular rash, etiological investigations were ordered, and topical methylprednisolone was initiated. The results of the investigations requested for etiologies that may cause a maculopapular rash are summarized in Table 1 and shown in Figure 1 and Figure 2.

By the end of the first week of hospitalization, acute-phase reactants had further increased, and, due to the persistence of febrile fever, piperacillin-tazobactam was added to the treatment regimen. Blood cultures yielded *Staphylococcus caprae*, and antimicrobial therapy was adjusted to piperacillin-tazobactam 4.5 g intravenously three times daily plus teicoplanin 400 mg intravenously once daily (with the first three doses administered every 12 hours). Echocardiography was performed to rule out infective endocarditis, and no findings suggestive of infective endocarditis were detected. At the end of the first week of hospitalization, the patient was re-consulted to the hematology department with a preliminary diagnosis of HLH due to persistent fever and elevated acute-phase reactants despite antibiotic therapy, bicytopenia, splenomegaly, hyperferritinemia, and hypofibrinogenemia.

**Figure 3:**
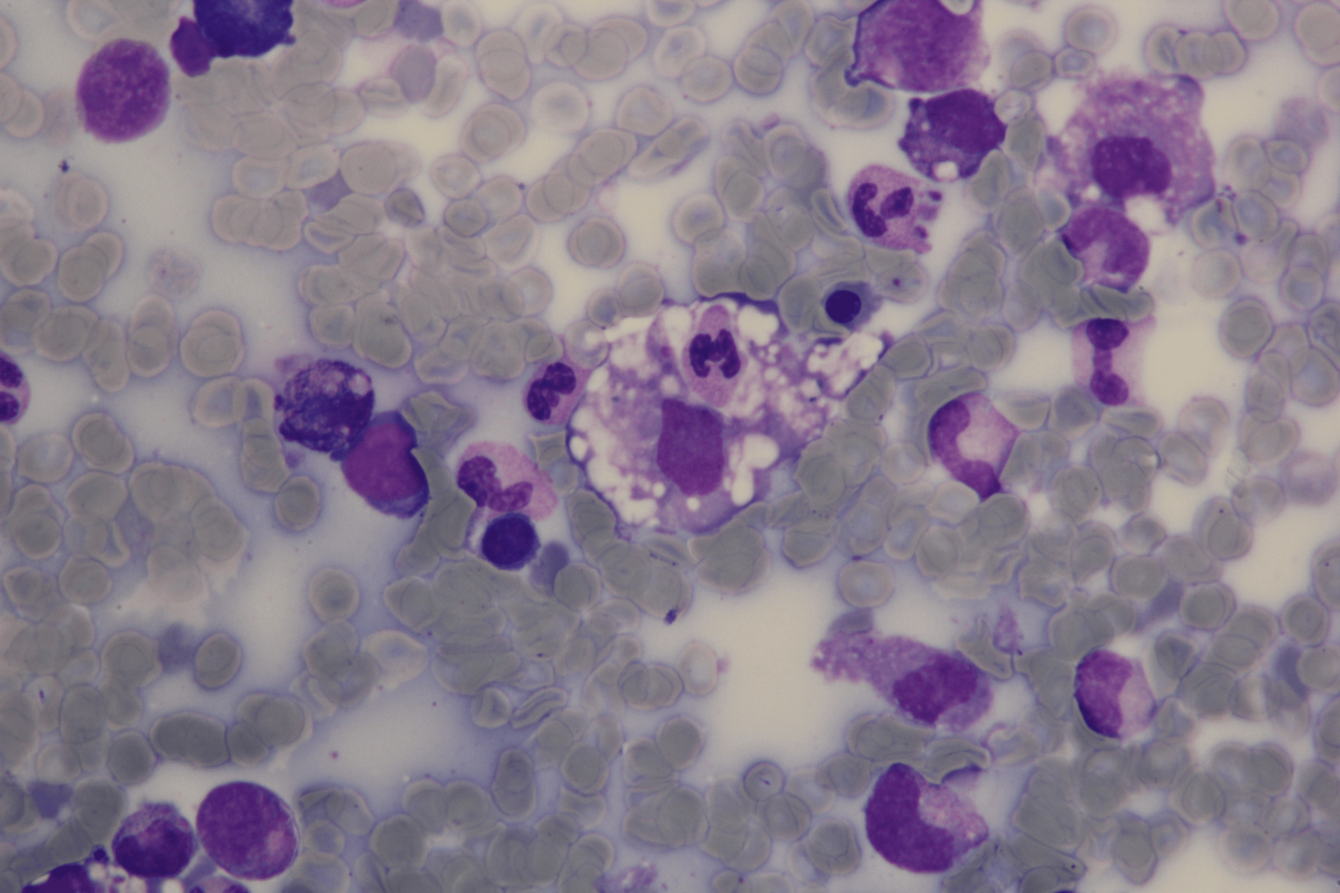
Bone marrow aspirate demonstrating hemophagocytosis. A macrophage containing phagocytosed nucleated cells and neutrophils within its cytoplasm is observed (original magnification ×1000, May-Grünwald-Giemsa).

On the 10th day, a punch biopsy and direct immunofluorescence were performed on the rash, and intravenous acyclovir was initiated. The biopsy findings were consistent with a drug eruption, and C3 deposition in the dermis was observed. Rheumatology evaluated the patient for adult-onset Still's disease and connective tissue disorders, recommending a bone marrow biopsy. Bone marrow findings were not consistent with HLH as shown in Figure 3.

On the 23rd day, dermatology and infectious diseases consultations were obtained, and investigations for various infectious diseases were requested, with results summarized in Table 1. Blood cultures were negative, although C-reactive protein (CRP) continued to rise (156 mg/L). On the 25th day, tigecycline therapy was initiated.

On the 27th day of hospitalization, further elevation of acute-phase reactants and persistence of fever prompted the initiation of vancomycin 1 g intravenously twice daily and etoposide 150 mg/m² with dexamethasone 16 mg for a preliminary diagnosis of HLH based on the HLH-2004 criteria shown in Table 2. The following day, the patient developed metabolic acidosis with abdominal breathing. Intensive care consultation was obtained based on arterial blood gas analysis; however, ICU admission was not recommended at that time. On the 29th day of hospitalization, intravenous immunoglobulin therapy was initiated for two days upon the recommendation of the hematology department due to a decrease in platelet count to 4,000/µL.

**Table 2:** HLH-2004 diagnostic criteria fulfilled by the patient. HLH: hemophagocytic lymphohistiocytosis

HLH-2004 criteria	Patient findings (with laboratory values)	Criteria met
Fever ≥38.5°C	Persistent febrile episodes during hospitalization	Yes
Splenomegaly	Hepatosplenomegaly detected on abdominal CT imaging	Yes
Cytopenia affecting ≥2 lineages	Hemoglobin: 7.5 g/dL; platelet count: 84×10⁹/L; white blood cell count: 8.32×10⁹/L (bicytopenia: anemia+thrombocytopenia)	Yes
Hypertriglyceridemia ≥265 mg/dL and/or hypofibrinogenemia ≤150 mg/dL	Triglycerides: 298 mg/dL	Yes
Hemophagocytosis in the bone marrow/spleen/lymph node	Bone marrow aspirate demonstrating hemophagocytosis	Yes
Low or absent NK cell activity	Not assessed	Not evaluated
Ferritin ≥500 ng/mL	Ferritin: 752 ng/mL	Yes
Elevated soluble CD25 (sIL-2 receptor)	Not assessed	Not evaluated

**Figure 4:**
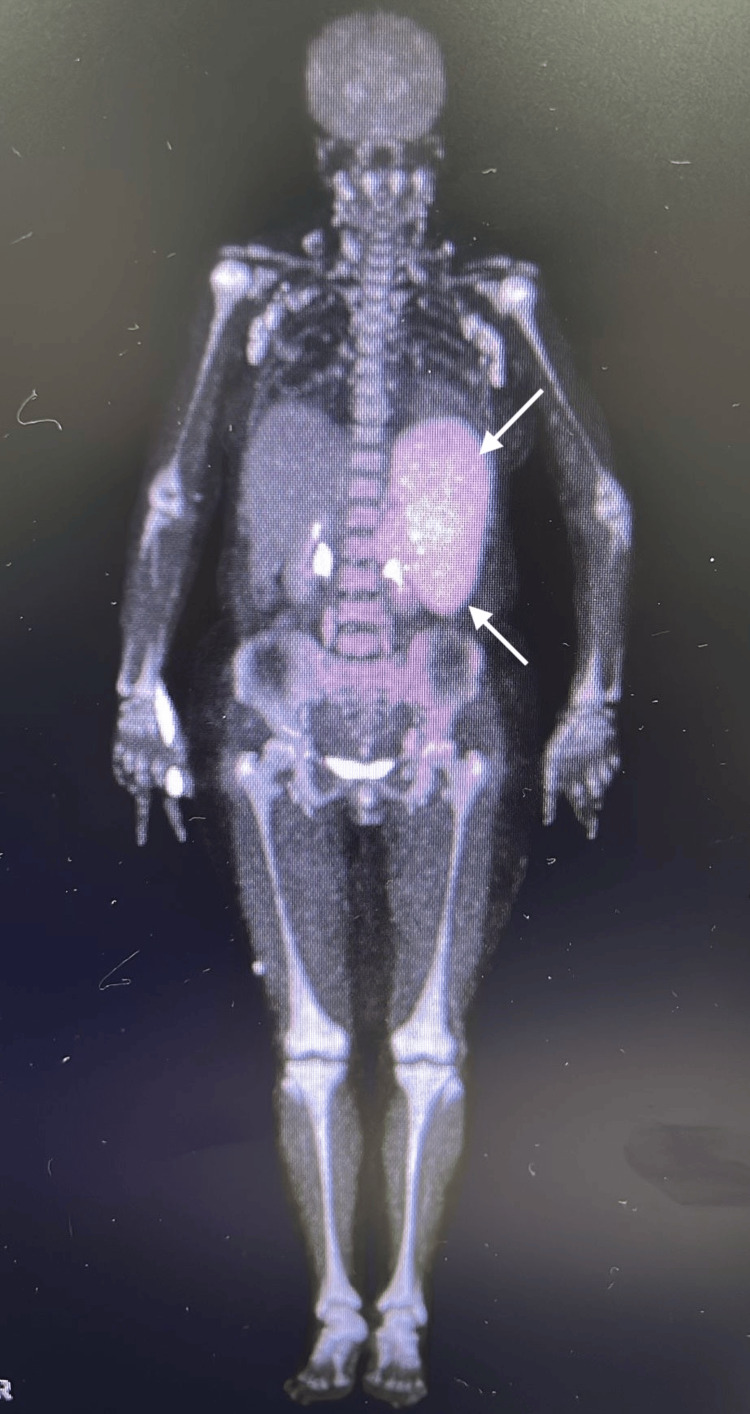
PET-CT showing splenomegaly. PET-CT: positron emission tomography-computed tomography

**Figure 5:**
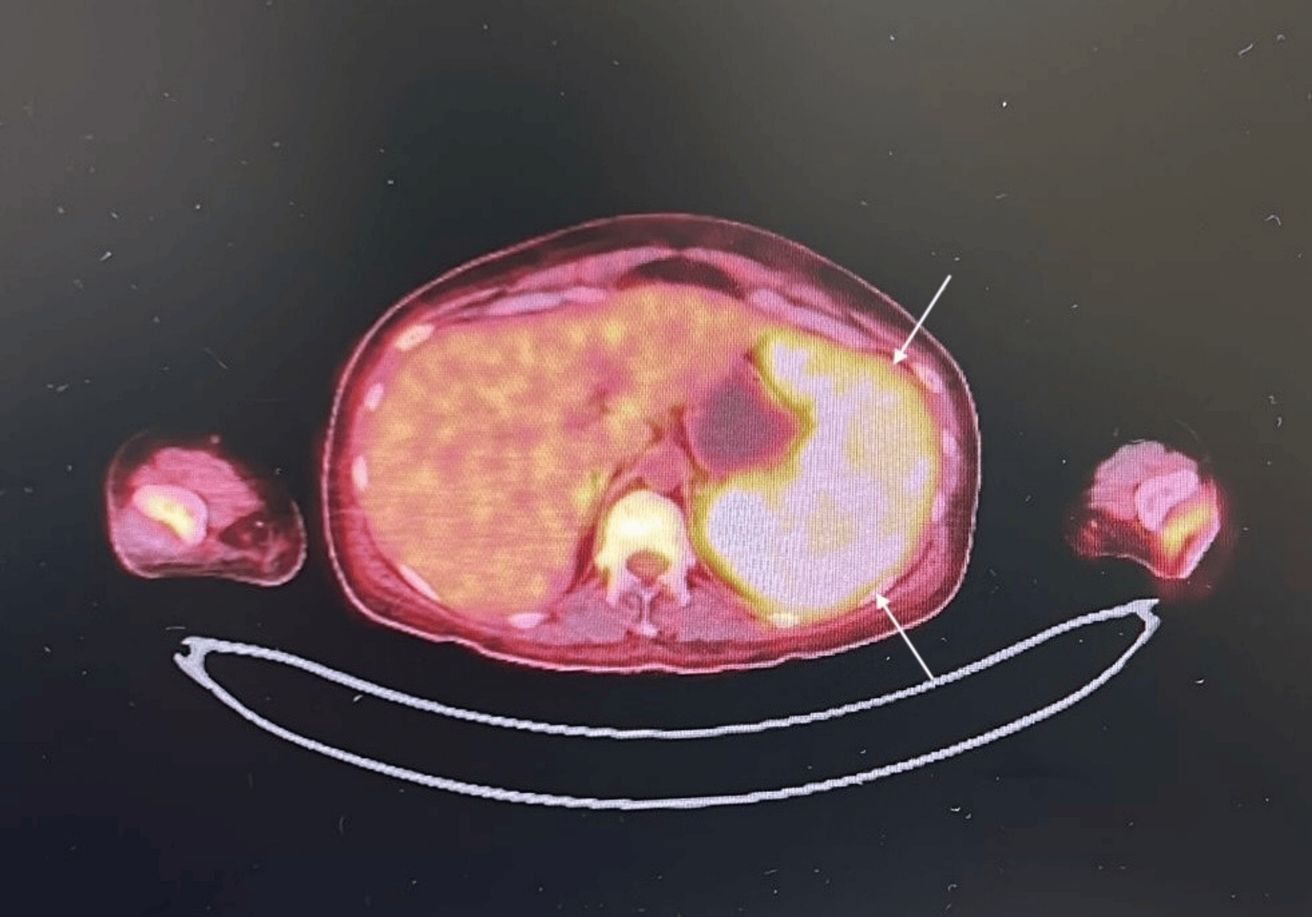
Diffuse splenic involvement in PET-CT. PET-CT: positron emission tomography-computed tomography

On the 34th day of hospitalization, positron emission tomography (PET) imaging performed for suspected macrophage activation syndrome revealed hypermetabolic nodular lesions with preserved fatty hilum and cortical thickening in both axillary regions. Excisional biopsy of the right axillary lymph node was recommended, and superficial ultrasonography was planned prior to the procedure as shown in Figure 4 and Figure 5.

On the 36th day, the patient was transferred to the hematology service for isolation and further evaluation of suspected acquired HLH. Prior to transfer, serological tests for infectious diseases are summarized in Table 1, and autoimmune and other disease markers, including tumor markers, imaging, and biopsy results, are summarized in Table 3.

**Table 3:** Autoimmune, rheumatologic, and malignancy workup. Anti-dsDNA: anti-double-stranded deoxyribonucleic acid; ENA: extractable nuclear antigen; p-ANCA: perinuclear anti-neutrophil cytoplasmic antibody; c-ANCA: cytoplasmic anti-neutrophil cytoplasmic antibody; IgM: immunoglobulin M; IgG: immunoglobulin G; CA-125: cancer antigen 125; CA 19-9: carbohydrate antigen 19-9; PET-CT: positron emission tomography-computed tomography; CT: computed tomography; FISH: fluorescence in situ hybridization; C3: complement component 3; IgA: immunoglobulin A

Investigation	Result	Reference range
Antinuclear antibody	Negative	<1/80
Anti-dsDNA	Negative	<25 IU/mL
ENA panel	Negative	<20 IU/mL
Anti-mitochondrial antibody	Negative	<20 IU/mL
Anti-smooth muscle antibody	Negative	<20 IU/mL
Anti-liver kidney microsomal antibody	Negative	<20 IU/mL
Rheumatoid factor	Normal	<14 IU/mL
Anti-cyclic citrullinated peptide	Negative	<20 IU/mL
p-ANCA	Negative	<20 IU/mL
c-ANCA	Negative	<20 IU/mL
Anti-cardiolipin IgM/IgG	Negative	<20 U/mL
Cryoglobulin	Negative	-
CA-125	42.1 U/mL	<35 U/mL
Carcinoembryonic antigen	Normal	<3 ng/mL
CA 19-9	Normal	<37 U/mL
Alpha-fetoprotein	Normal	<10 ng/mL
Albumin/creatinine ratio (spot urine)	39 mg/g (microalbuminuria)	<30 mg/g
PET-CT	Diffuse hypermetabolic splenic, hepatic, skeletal, and nodal involvement	-
Thoracic CT and angiography	No findings suggestive of malignancy or vasculitis	-
Abdominal CT and angiography	Hepatosplenomegaly was present, with no findings suggestive of vasculitis	-
Bone marrow flow cytometry	No diagnostic clonal population	-
FISH panel	No rearrangement detected	-
Skin biopsy (direct immunofluorescence)	Fibrinogen and C3 deposition; IgA/IgG/IgM negative	-
Excisional lymph node biopsy	Kaposi sarcoma, extramedullary hematopoiesis	-

Following the patient's admission to the hematology ward, a tru-cut biopsy was planned from the right axillary lymph node identified on PET as having the most superficial involvement, and the result was reported as non-diagnostic. Given the clinical suspicion and the similarity of the current presentation to hematologic-oncologic diseases, an excisional axillary lymph node biopsy was subsequently decided upon.

**Figure 6:**
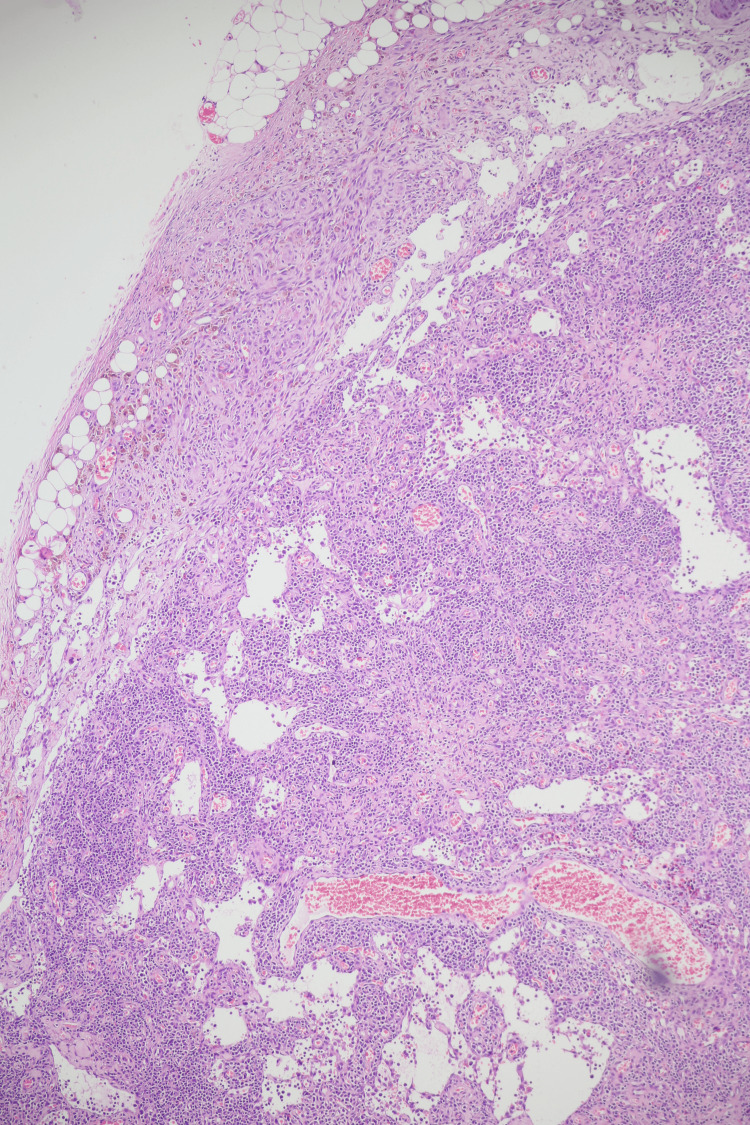
Excisional biopsy of the right axillary lymph node showing a neoplastic spindle cell proliferation within the capsular and pericapsular regions. The tumor cells form short fascicles and are associated with slit-like vascular spaces and extravasated erythrocytes (original magnification ×100, hematoxylin and eosin).

**Figure 7:**
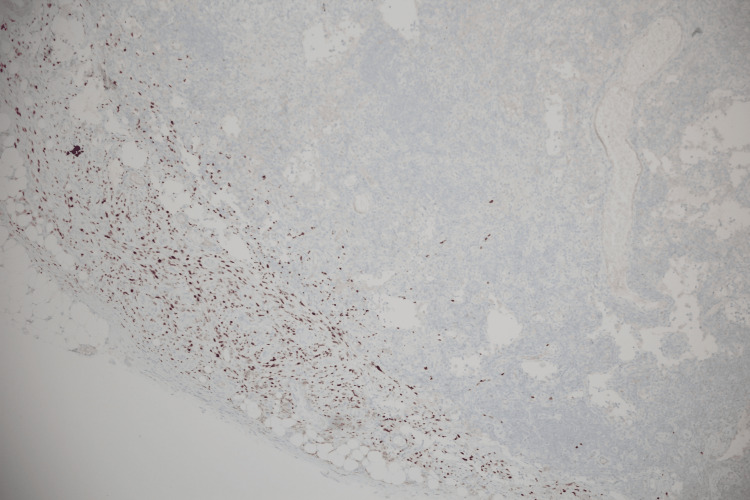
Diffuse and strong nuclear positivity for HHV-8 (LNA-1) within the spindle cell proliferation, supporting the diagnosis of Kaposi Sarkomu (Kaposi sarcoma) (original magnification ×100, immunohistochemistry). HHV-8: human herpesvirus 8; LNA-1: latent nuclear antigen-1

During hospitalization, the patient received etoposide and dexamethasone at appropriate doses for eight weeks for the treatment of HLH. During the course of admission to the hematology service, the pathology result of the excisional lymph node biopsy was reported as consistent with KS, as shown in Figure 6. Also, immunohistochemical staining showed diffuse nuclear positivity for human herpesvirus 8 (latent nuclear antigen-1 (LNA-1)), confirming the diagnosis shown in Figure 7.

Following the histopathological confirmation of KS, the patient was evaluated by the oncology department. Paclitaxel therapy was initiated at a dose of 100 mg/m² administered over three hours every two weeks in accordance with the European consensus-based interdisciplinary guideline for the diagnosis and treatment of KS (European Dermatology Forum/European Association of Dermato-Oncology/European Organization for Research and Treatment of Cancer (EDF/EADO/EORTC)), where paclitaxel is recommended as a first-line systemic therapy for advanced or visceral disease. After two weeks of treatment, the patient's general condition improved, and HLH therapy was planned to be completed over an eight-week period; the patient was subsequently transferred to the oncology service, and the regimen was modified to include etoposide plus paclitaxel. The patient remained hospitalized in the oncology service for two weeks and was discharged with a plan for outpatient paclitaxel therapy.

Overall, the patient was hospitalized for 112 days and is currently followed in the outpatient oncology clinic, continuing paclitaxel therapy (100 mg/m²). To date, no complications have occurred, and follow-up imaging has demonstrated the regression of hepatosplenomegaly (Table 4).

**Table 4:** CARE timeline table. PET-CT: positron emission tomography-computed tomography; HLH: hemophagocytic lymphohistiocytosis; CARE: Case REport

Time	Key clinical events
Week 1	Etiologies of hepatosplenomegaly, fever, rash, and bicytopenia were investigated. Infectious serology, endoscopy/colonoscopy, and peripheral smear were performed; treatment was arranged with infectious diseases and dermatology
Week 2	Persistent fever and elevated inflammatory markers despite treatment. Extensive infectious, autoimmune, and hematologic investigations were performed. Skin biopsy revealed a drug eruption. Hematology and rheumatology consultations were made
Weeks 3-4	Continued clinical deterioration with persistent fever and cytopenias. Secondary HLH was suspected and diagnosed, and etoposide plus dexamethasone therapy was initiated
Week 5	PET-CT demonstrated hypermetabolic bilateral axillary lymphadenopathy with splenic involvement
Week 6	Excisional axillary lymph node biopsy was performed after a non-diagnostic tru-cut biopsy
Weeks 7-8	Pathology confirmed Kaposi sarcoma. HLH therapy was continued
Weeks 9-12	The patient was transferred to the oncology service, and treatment was changed to etoposide plus paclitaxel. Clinical condition gradually stabilized
Week 13	The patient was discharged with planned outpatient weekly paclitaxel therapy and oncology follow-up

## Discussion

In the etiology of FUO, infectious diseases, malignancies, collagen tissue diseases, vasculitis, and autoimmune diseases, as well as inflammatory and autoinflammatory disorders, should be considered [5].

The causes of FUO have been reported in the literature to be influenced by the economic status of the countries in which patients live. Since our patient resided in Türkiye, we initially focused on etiologies more prevalent in low- and middle-income settings. In one study, recent FUO reports from low- and middle-income countries showed that infections constituted the majority of cases at 43-63%, followed by neoplasms at 1-22%, collagen tissue diseases at 13-30%, various other diseases at 2-14%, and undiagnosed FUO cases at 2-12%. In contrast, studies from middle- and high-income countries reported the distribution as follows: infections 15-49%, neoplasms 7-18%, collagen tissue diseases 19-47%, various other diseases 1-13%, and undiagnosed cases 8-30% [6].

HLH is a life-threatening condition characterized by a cytokine storm resulting from the dysregulated activation and proliferation of natural killer cells, CD8+ cytotoxic T lymphocytes, and macrophages. These cells are key components of cellular immunity responsible for eliminating target cells; however, their uncontrolled activation leads to systemic inflammation, increased phagocytic activity-associated tissue damage, multiple organ failure, and death. HLH is classified into two forms: a primary form resulting from inherited genetic mutations and a secondary form that may develop due to infections, malignancies, or autoimmune diseases. Primary HLH typically presents in childhood, whereas secondary HLH is more commonly observed in adults, usually in the setting of an acute illness and most frequently associated with infectious diseases or hematologic malignancies [7].

In our case, secondary HLH associated with KS was considered highly likely to lead to death due to profound cytopenias, cytokine storm, and sepsis if not promptly controlled with dexamethasone and etoposide therapy. The literature also includes studies focusing on the identification of prognostic markers for this fatal condition; one study reported that age, lactate dehydrogenase (LDH) level, and platelet count are important prognostic parameters [8].

Although our patient showed weakly positive low-titer seropositivity on the dipstick test and *Leishmania* spp. immunoglobulin G (IgG) positivity at a titer of 1:160 by indirect fluorescent antibody test (IFAT), previous studies have reported that certain infections, autoimmune diseases, malignancies, and the technical limitations of antibody-based assays such as IFAT may lead to false-positive results due to cross-reactivity, particularly in immunocompetent patient populations [9,10]. In our patient, multiple consultations with the infectious diseases department determined that *Leishmania* was an unlikely cause of the clinical picture, and it was therefore decided that hematologic-oncologic disorders should be definitively ruled out by excisional lymph node biopsy.

Multidisciplinary council evaluations conducted during the investigation of FUO enable the timely establishment of a definitive diagnosis without delay. In our patient, infectious etiologies were initially considered; however, advanced diagnostic evaluations ultimately led to the diagnosis of KS.

In conclusion, KS is a vascular tumor that may be fatal if left untreated. It can present with HLH and, although rare, should be considered in the etiology of FUO, requiring rapid diagnosis and the prompt initiation of treatment.

## Conclusions

KS can present with atypical systemic manifestations and may rarely trigger secondary HLH, creating significant diagnostic challenges. In patients presenting with FUO, cytopenias, and hepatosplenomegaly who do not respond to initial treatment, underlying malignancy should be carefully considered. This case highlights the importance of a multidisciplinary diagnostic approach and timely tissue biopsy to establish the correct diagnosis. Recognition of the underlying cause of HLH is important for guiding appropriate diagnostic evaluation and therapeutic decision-making, which may contribute to improved clinical management.
